# Supplementary material for: Architecture of a complete Bce-type antimicrobial peptide resistance module
Source: Nat Commun. 2023 Jul 1;14:3896. doi: 10.1038/s41467-023-39678-w (PMC10314905; doi:10.1038/s41467-023-39678-w)
Supplement: Supplementary file 1 — Supplementary Information [file 41467_2023_39678_MOESM1_ESM.pdf]

Figure S1: Biochemical characterization of BceAB-S

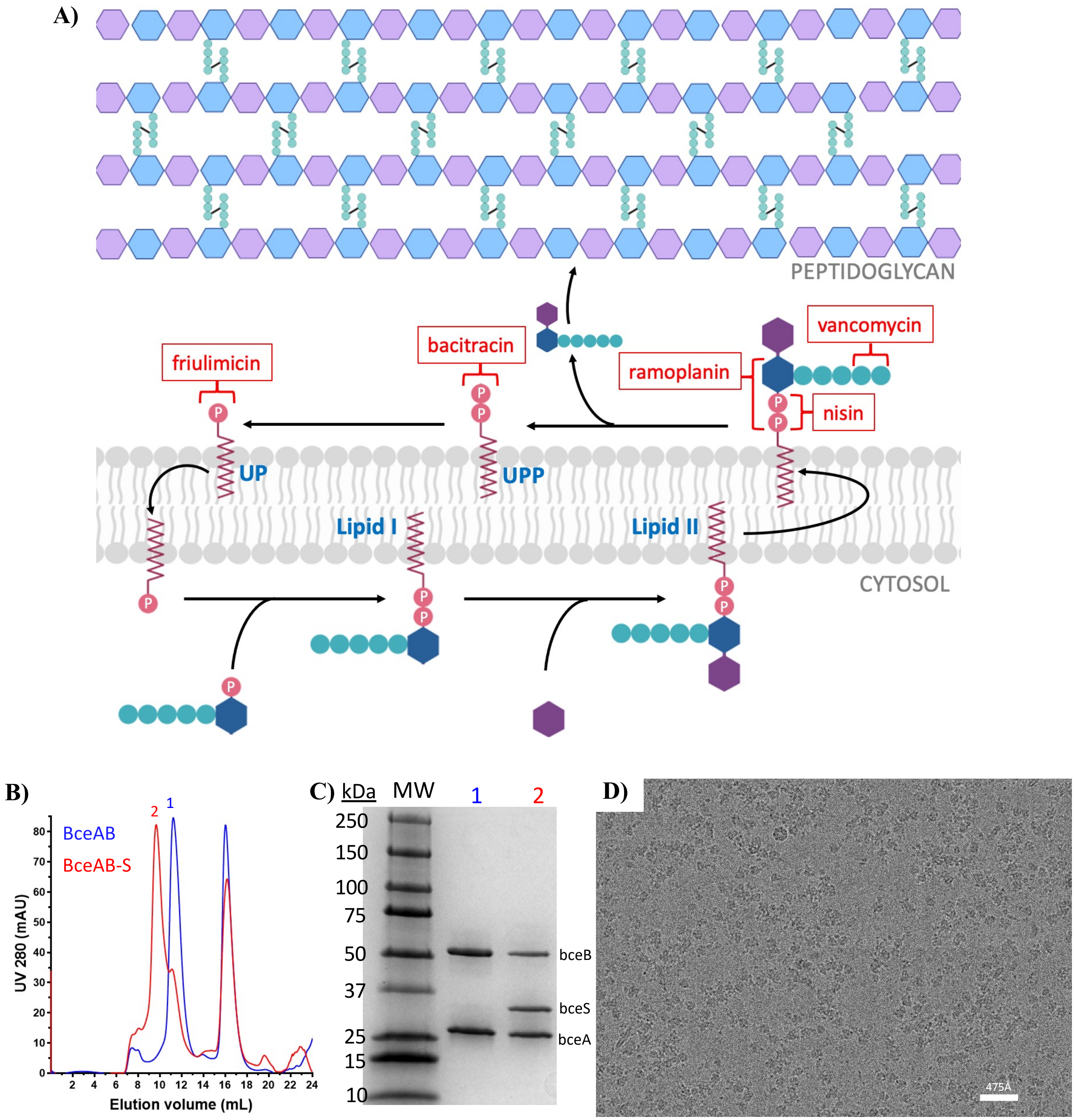

Figure S1: Biochemical characterization of BceAB-S

A) Schematic showing the lipid II cycle of peptidoglycan synthesis. Lipid intermediates are indicated in blue letters. (UPP: undecaprenyl pyrophosphate, UP: undecaprenyl phosphate). Antimicrobial peptides are indicated in red boxes, with brackets indicating the motifs they recognize on individual lipid intermediates. **B)** Gel-filtration profiles of detergent solubilized BceAB (blue trace) and BceAB-S (red trace) complexes. Numbers indicate peaks from each gel-filtration run that were analyzed via SDS-PAGE. **C)** SDS-PAGE analysis of the gel-filtration peaks indicated in **B**, demonstrating that the shift to higher molecular weight observed with BceAB-S is due to the presence of BceS. This experiment has been repeated twice with similar results. **D)** Electron micrograph of detergent solubilized BceAB-S complexes showing a uniform particle distribution in thin ice. The white scale bar demonstrates a size of 475Å. Approximately ~9000 similar micrographs were obtained.

Figure S2: Cryo-EM Data Processing for nucleotide-free BceAB-S

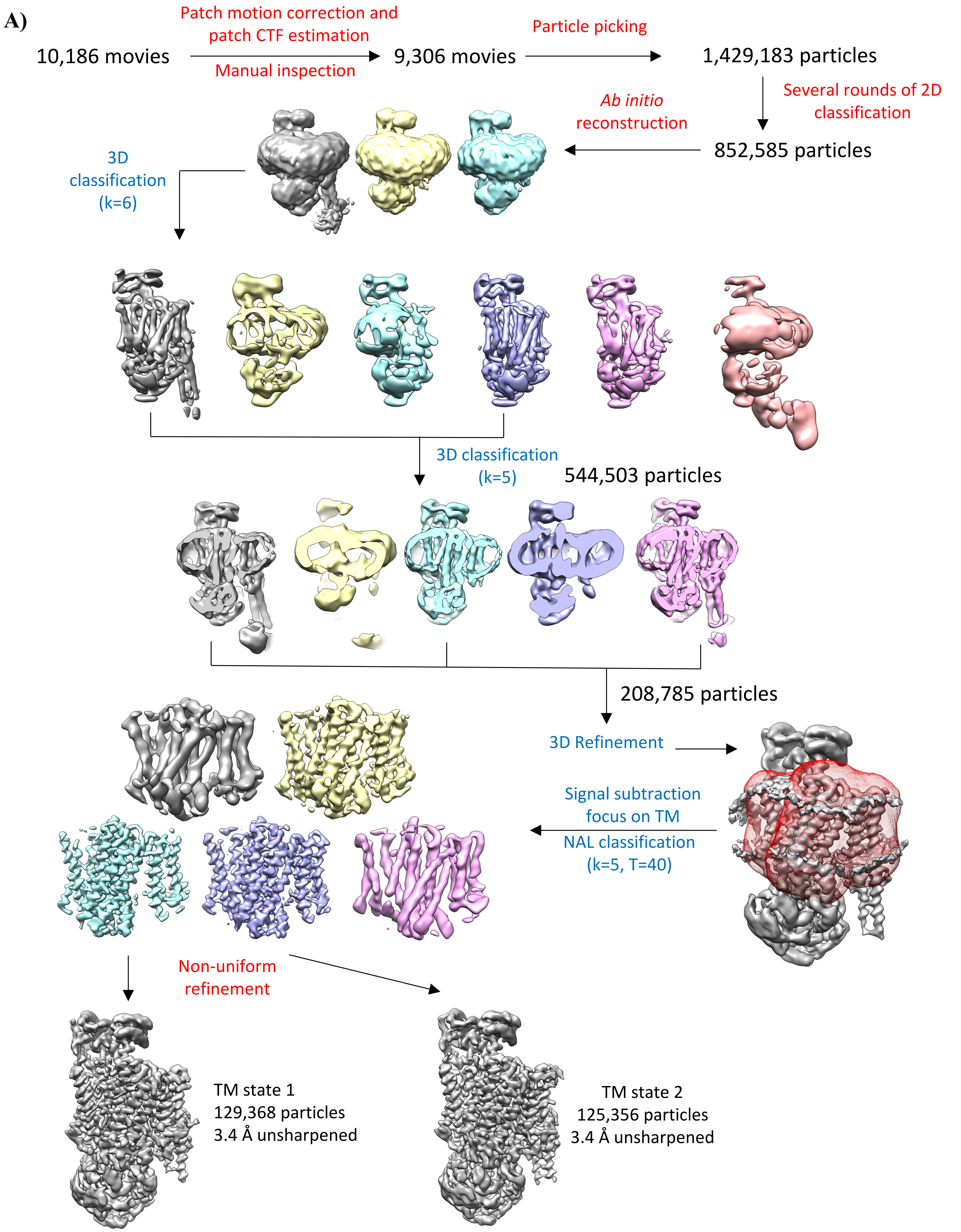

Figure S2: Cryo-EM Data Processing for nucleotide-free BceAB-S

A) Data processing to resolve high resolution features in the TM region of nucleotide-free BceAB-S. Steps indicated in red were performed in CryoSPARC, and steps in blue were performed in Relion.

Figure S3: Cryo-EM Data Analysis of nucleotide-free BceAB-S TM State 1

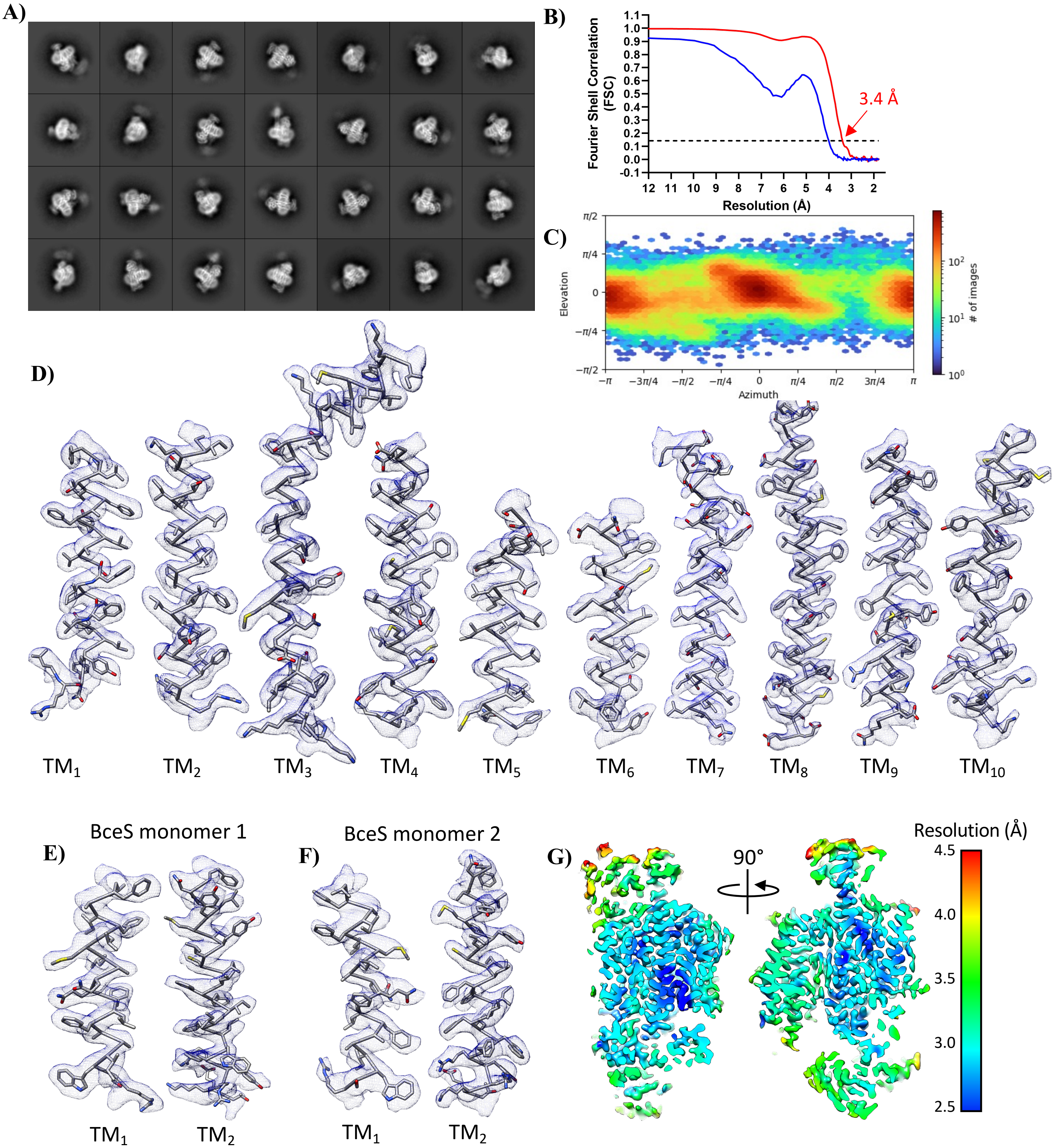

Figure S3: Cryo-EM Data Analysis of nucleotide-free BceAB-S TM State 1

A) 2D class-averages of nucleotide-free BceAB-S particles. Flexibility of the BceS soluble domains is evident in most 2D averages. **B)** Fourier shell correlation (FSC) curve for the final nucleotide-free BceAB-S TM state 1 reconstruction. Blue curve was generated without masking, and the red curve was generated with a tight auto-mask in CryoSPARC. Gold-standard FSC cutoff of 0.143 is indicated with a dashed black line. **C)** Angle distribution of particles in the final reconstruction. **D-F)** Experimental cryo-EM map (transparent blue mesh) and fitted atomic model for TM helices throughout the BceAB-S complex. **G)** Local resolution map indicating varying resolution throughout the cryo-EM map.

Figure S4: Cryo-EM Data Analysis of nucleotide-free BceAB-S TM State 2

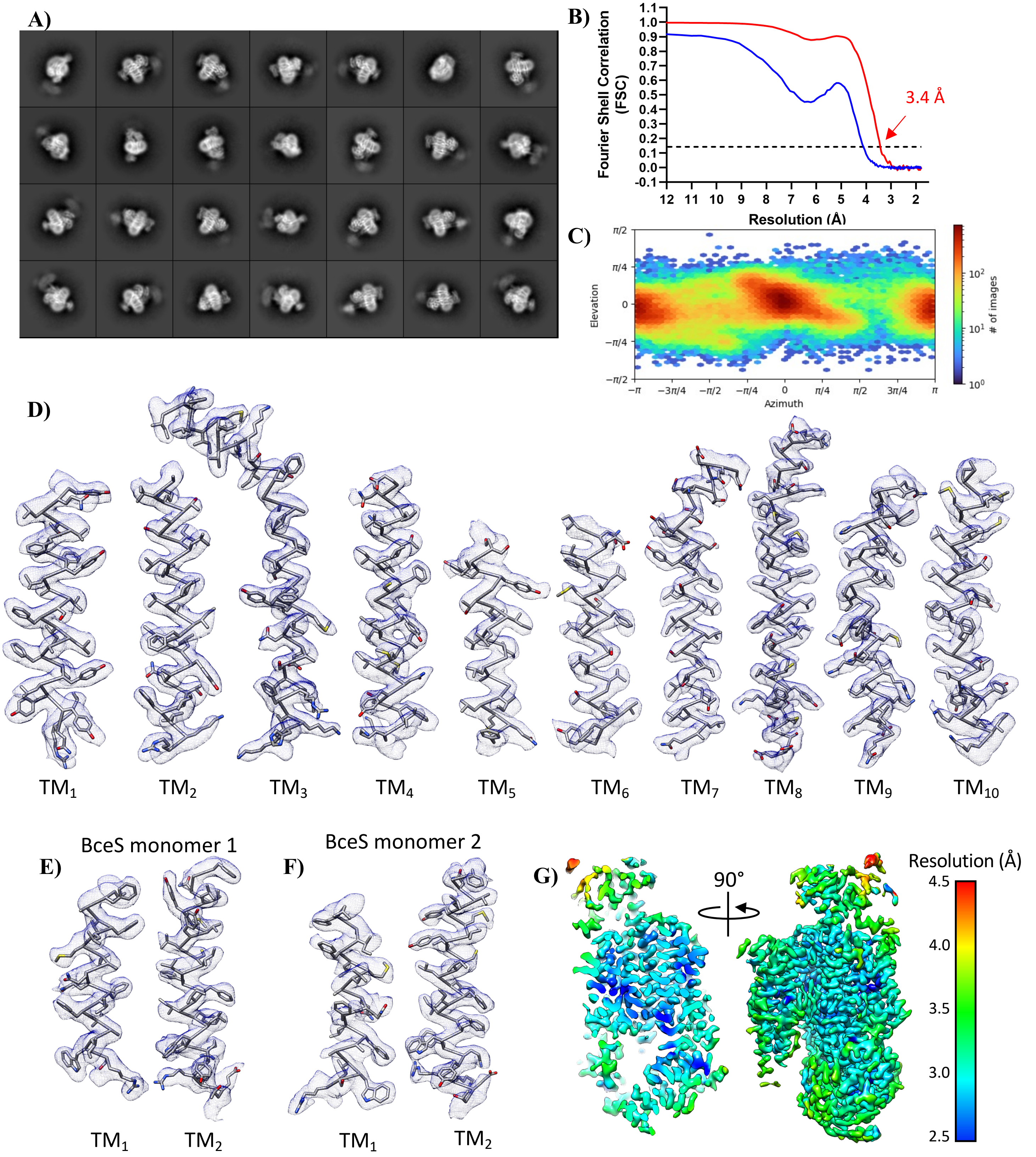

Figure S4: Cryo-EM Data Analysis of nucleotide-free BceAB-S TM State 2

A) 2D class-averages of nucleotide-free BceAB-S particles. Flexibility of the BceS soluble domains is evident in most 2D averages. **B)** FSC curve for the final nucleotide-free BceAB-S TM state 2 reconstruction. Blue curve was generated without masking, and the red curve was generated with a tight auto-mask in CryoSPARC. Gold-standard FSC cutoff of 0.143 is indicated with a dashed black line. **C)** Angle distribution of particles in the final reconstruction. **D-F)** Experimental cryo-EM map (transparent blue mesh) and fitted atomic model for TM helices throughout the BceAB-S complex. **G)** Local resolution map indicating varying resolution throughout the cryo-EM map.

Figure S5: Comparison of BceS to Other Histidine Kinases

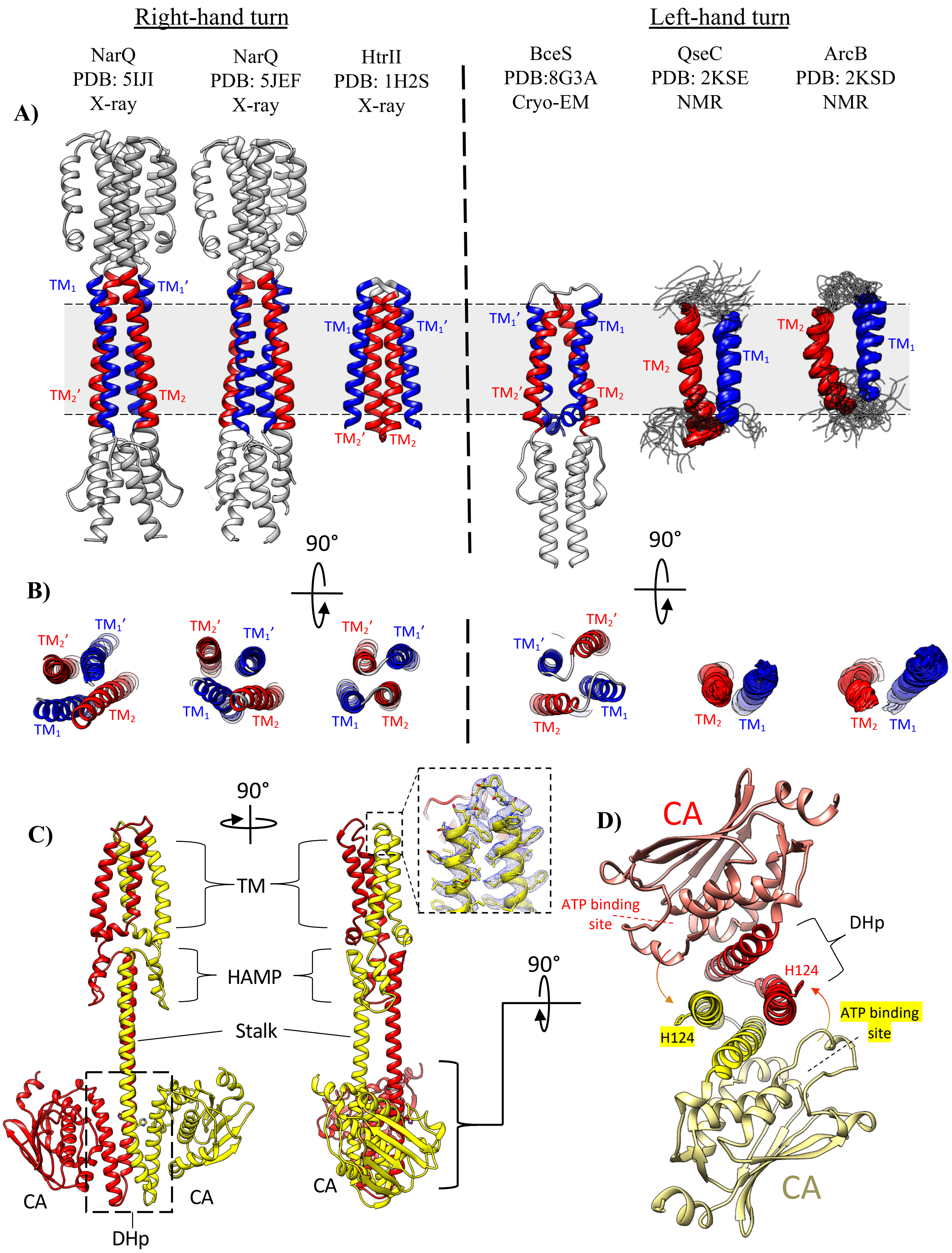

Figure S5: Comparison of BceS to Other Histidine Kinases
A) Comparison of the TM helix arrangement in BceS versus other histidine kinases of known structure. TM_1 is colored blue and TM_2 is colored red in all structures. **B)** 90° rotated view of **A** showing TM helix configuration in all structures. **C)** Domain architecture of BceS. Individual BceS monomers are shown in red or yellow cartoon, with domains highlighted with black letters. The inset outlined in dashed black lines shows the cryo-EM map of the TM_1-TM_2 linker in BceS. **D)** View from the membrane of the DHp and CA domains in BceS. The autophosphorylated H124 residue in the DHp domain and the ATP binding sites in the CA domains are indicated. The configuration of domains in BceS indicate that autophosphorylation occurs in *trans* (indicated by arrows between ATP binding site and phosphorylated H124).

Figure S6: Cryo-EM Data Processing of Nucleotide-Free BceAB-S to Reveal BceS

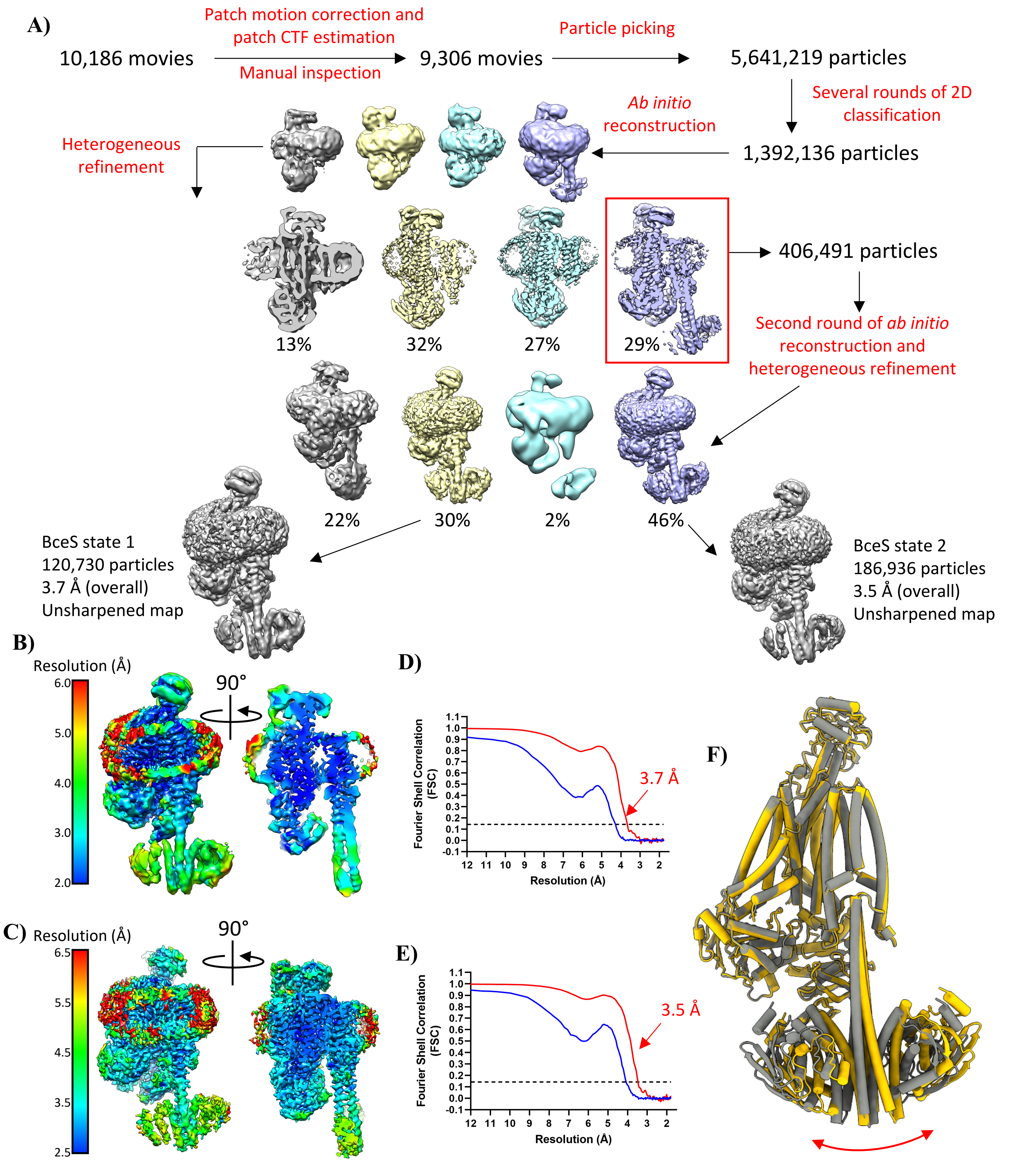

Figure S6: Cryo-EM Data Processing of Nucleotide-Free BceAB-S to Reveal BceS
A) Cryo-EM data processing workflow to resolve the entirety of BceS in the nucleotide-free BceAB-S complex. Steps indicated in red were performed in CryoSPARC, and steps indicated in blue were performed in Relion. **B&C)** Local resolution map indicating varying resolution throughout the cryo-EM model for BceS state 1 (**B**) and BceS state 2 (**C**). **D&E)** FSC curve for BceS state 1 (**D**) and BceS state 2 (**E**). Blue curves were generated without masking, and the red curve was generated with a tight auto-mask in CryoSPARC. Gold-standard FSC cutoff of 0.143 is indicated with a dashed black line **F)** Comparison of BceS state 1 and state 2 indicating alternate conformations of the DHp/CA domain in BceS. Red arrow indicates difference in movement of BceS between the two reconstructions.

Figure S7: Cryo-EM Data Processing for ATPγS bound BceAB-S

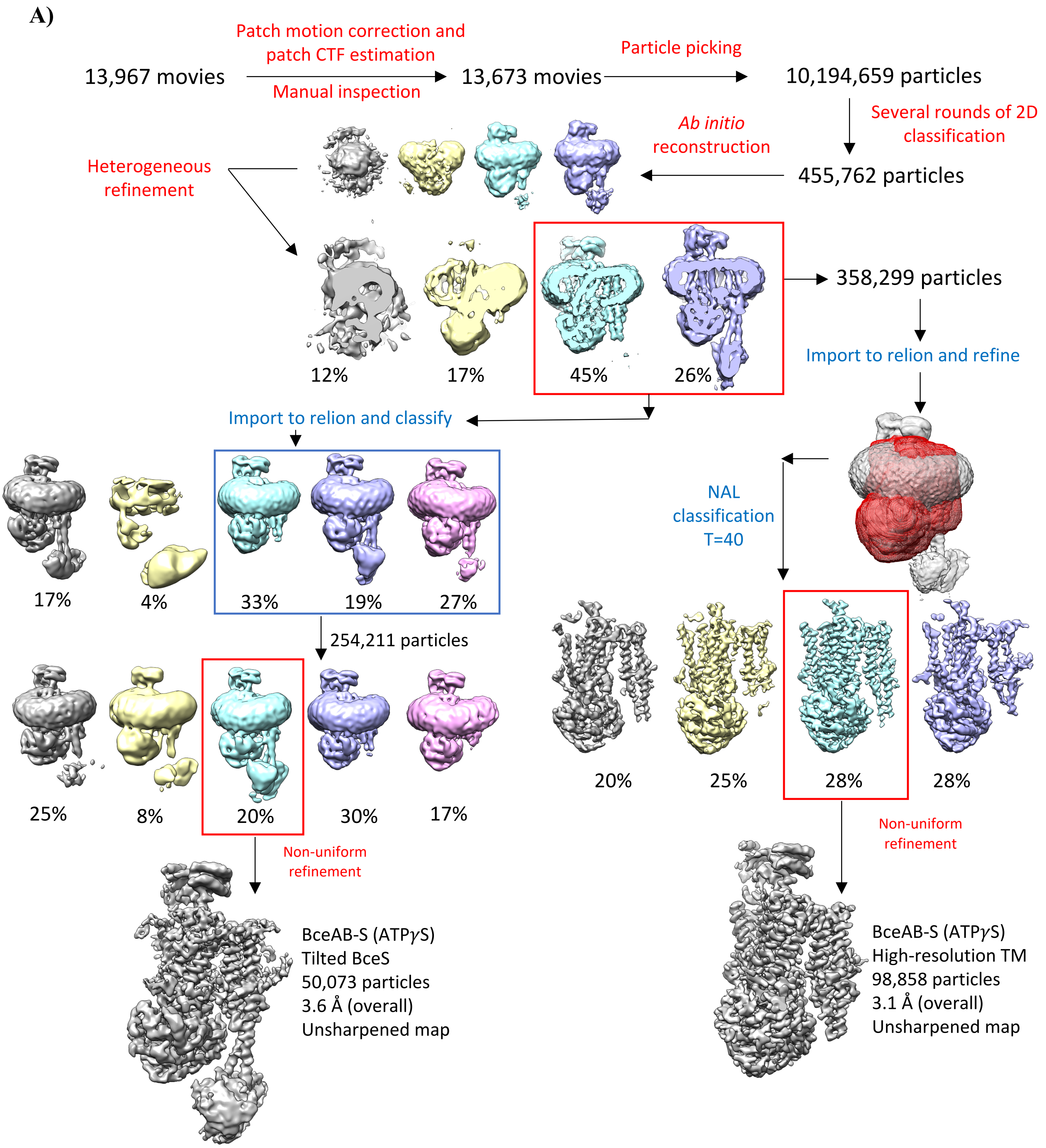

Figure S7: Cryo-EM Data Processing for ATPγS bound BceAB-S

A) Cryo-EM data processing workflow to resolve two different states of the BceAB-S complex bound to the nucleotide analog ATPγS. One reconstruction produced high-resolution features throughout the TM region, and the second reconstruction at slightly lower overall resolution revealed the entirety of BceS. Steps indicated in red were performed in CryoSPARC, and steps indicated in blue were performed in Relion.

Figure S8: Cryo-EM Results for ATPγS bound BceAB-S

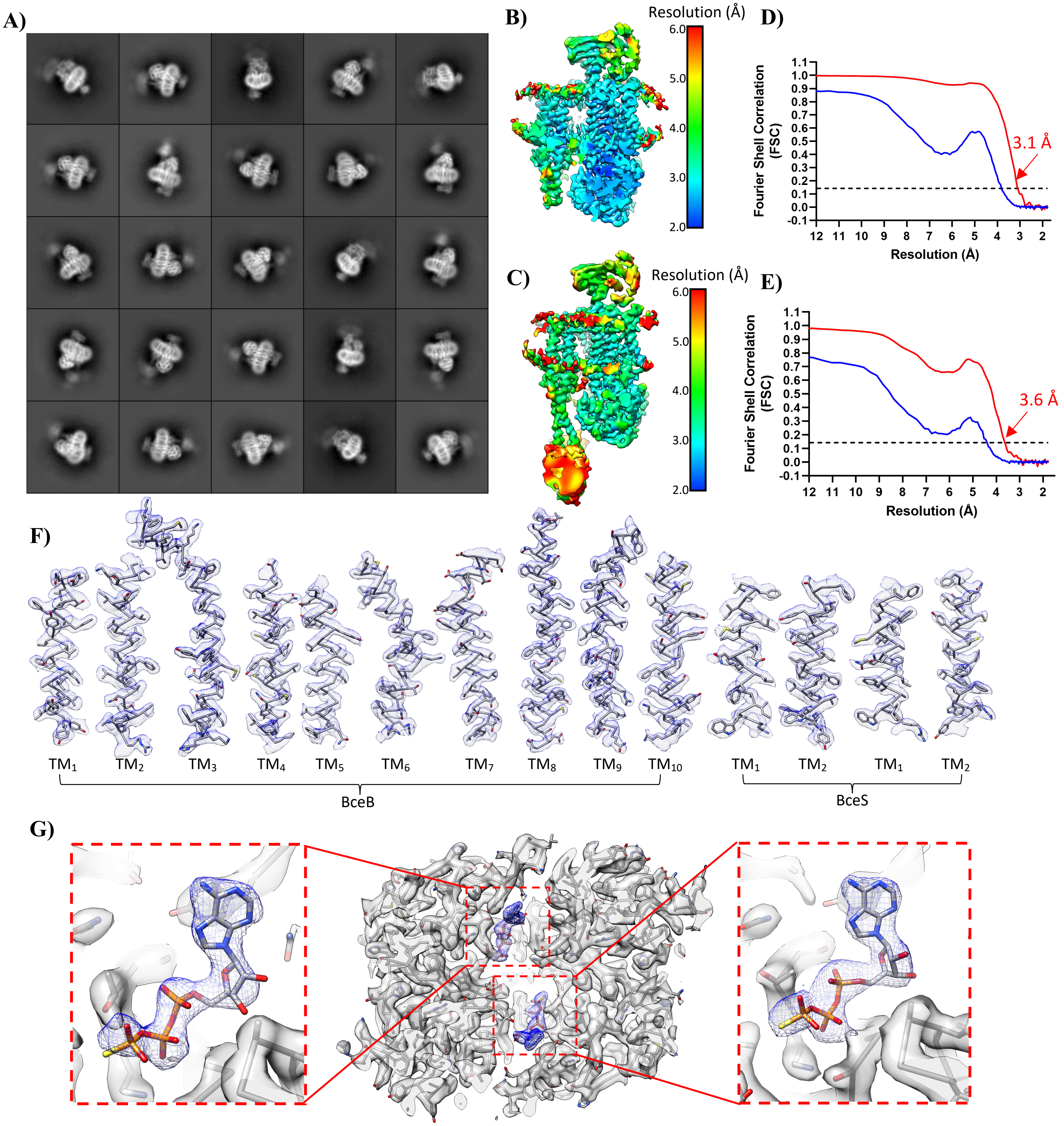

Figure S8: Cryo-EM Results for ATPγS bound BceAB-S

A) 2D class-averages of BceAB-S particles in the presence of ATPγS. **B&C)** Local resolution map indicating varying resolution throughout the cryo-EM map for the reconstruction with high-resolution TM features (**B**) or with tilted BceS (**C**). **D&E)** FSC curve for the reconstruction with high-resolution TM features (**D**) or with tilted BceS (**E**). Blue curves were generated without masking, and the red curve was generated with a tight auto-mask in CryoSPARC. Gold-standard FSC cutoff of 0.143 is indicated with a dashed black line **F)** Representative cryo-EM maps for the TM helices throughout the ATPγS bound BceAB-S complex. **G)** Cryo-EM map showing the presence of ATPγS (blue mesh) bound in the two potential ATP binding sites between monomers of BceA (transparent grey surface with grey sticks).

Figure S9: Proposed Model of BceAB-S Function

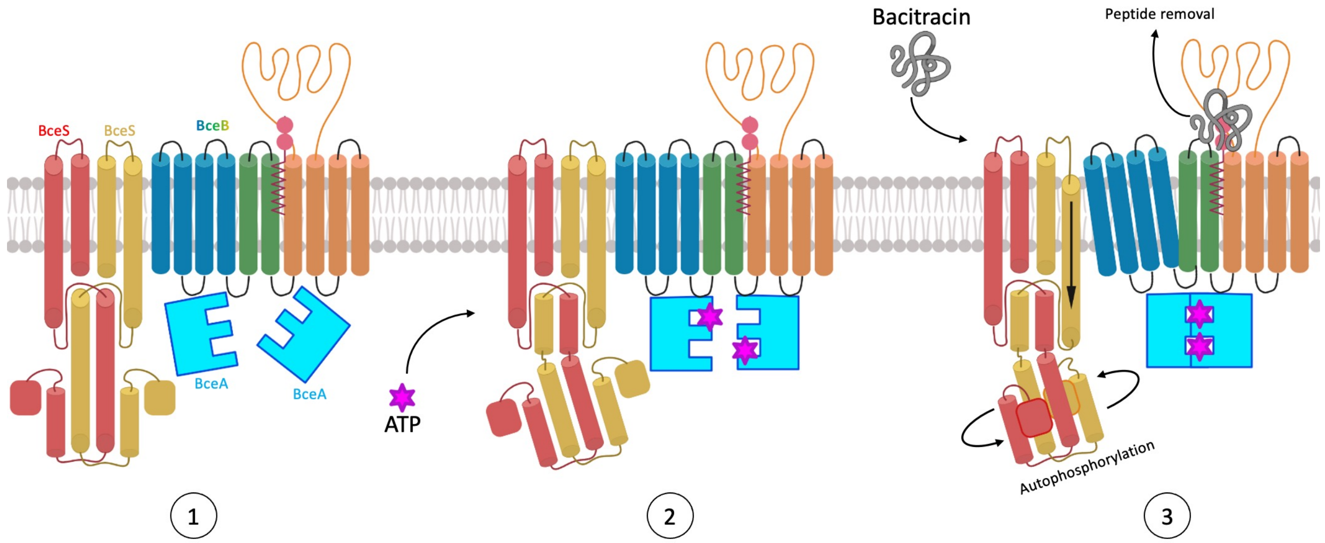

Figure S9: Proposed Model of BceAB-S Function

Cartoon diagram depicting a proposed model of steps involved in BceAB-S activation and signaling. In the resting nucleotide-free state (state 1) the BceA nucleotide binding domains adopt an asymmetric configuration. Subsequent binding of ATP (purple star) induces the BceA subunits to adopt a more symmetrical configuration (state 2), with each BceA subunit engaging ATP but not fully dimerized to support ATP hydrolysis. In state 2 the stalk helix connecting the HAMP and CA domains of BceS develops a kink. Subsequent binding of bacitracin (state 3) induces the BceA subunits to fully dimerize and initiate ATP hydrolysis, which induces a conformational change of the BceB TM helices that is in-turn sensed by BceS in the form of downward piston-like movement of BceS TM-2. The conformational change of BceS is propagated downward through the HAMP and DHp domains, resulting in rotation of the CA domain to support autophosphorylation. During this process bacitracin is removed from the lipid target undecaprenyl-pyrophosphate. ATP hydrolysis by BceA resets the complex to the resting nucleotide-free state.

Table S1: Cryo-EM data collection and processing statistics

	BceAB-S Nucleotide-free TM state-1 (EMDB-29690) (PDB 8G3A)	BceAB-S Nucleotide-free TM state-2 (EMDB-29691) (PDB 8G3B)	BceAB-S Nucleotide-free BceS state-1 (EMDB-29694) (PDB 8G3F)	BceAB-S Nucleotide-free BceS state-2 (EMDB-29701) (PDB 8G3L)	BceAB-S ATPγS-bound High-res TM (EMDB-29716) (PDB 8G4C)	BceAB-S ATPγS-bound Kinked BceS (EMDB-29717) (PDB 8G4D)
**Data collection and processing**						
Magnification	105,000	105,000	105,000	105,000	105,000	105,000
Voltage (kV)	300	300	300	300	300	300
Electron exposure (e–/Å^2)	60.5	60.5	60.5	60.5	60.5	60.5
Defocus range (μm)	0.7-2.5	0.7-2.5	0.7-2.5	0.7-2.5	0.7-2.5	0.7-2.5
Pixel size (Å)	0.872	0.872	0.872	0.872	0.872	0.872
Symmetry imposed	C1	C1	C1	C1	C1	C1
Initial particle images (#)	852,585	852,585	1,392,136	1,392,136	455,762	455,762
Final particle images (#)	129,368	125,346	120,730	186,936	98,858	50,073
Map resolution (Å)	3.4	3.5	3.7	3.5	3.1	3.6
FSC threshold	0.143	0.143	0.143	0.143	0.143	0.143
**Refinement**						
Model resolution (Å)	3.7	3.7	3.9	3.7	3.4	3.8
FSC threshold	0.5	0.5	0.5	0.5	0.5	0.5
Map sharpening *B* factor (Å^2)	-113	-110	-113	-110	-76	-67
Map-model CC						
CC_{mask}	0.71	0.74	0.72	0.73	0.77	0.73
CC_{box}	0.60	0.63	0.68	0.67	0.62	0.69
CC_{volume}	0.66	0.69	0.68	0.68	0.73	0.70
CC_{peaks}	0.51	0.53	0.56	0.58	0.55	0.56
Model composition						
Non-hydrogen atoms	14441	14394	14441	14441	14498	14397
Protein residues	1791	1791	1791	1791	1802	1795
Ligands	4	3	4	4	3	2
B factors (Å^2)						
Protein	38.8	47.93	48.9	32.4	56.7	61.7
Ligand	17.6	18.64	15.9	6.5	27.0	27.8
R.m.s. deviations						
Bond lengths (Å)	0.003	0.003	0.003	0.005	0.003	0.003
Bond angles (°)	0.544	0.538	0.611	0.655	0.479	0.653
Validation						
MolProbity score	1.75	1.69	1.84	1.80	1.63	1.96
Clashscore	6.37	5.82	8.08	7.67	6.03	10.57
Poor rotamers (%)	0.06	0.06	0.32	0.00	0.00	0.00
Ramachandran plot						
Favored (%)	94.04	94.55	94.04	94.49	95.69	93.66
Allowed (%)	5.85	5.45	5.96	5.51	4.31	6.34
Disallowed (%)	0.11	0.00	0.00	0.00	0.00	0.00
