# Supplementary material for: Architecture of a complete Bce-type antimicrobial peptide resistance module
Source: Nat Commun. 2023 Jul 1;14:3896. doi: 10.1038/s41467-023-39678-w (PMC10314905; doi:10.1038/s41467-023-39678-w)
Supplement: Supplementary file 3 — Description of additional supplementary files [file 41467_2023_39678_MOESM3_ESM.docx]

**Description of additional supplementary files**

**Title**: Movie S1

**Description**: Morph showing the small scale structural differences between TM state-1 and TM state-2 structures of the nucleotide-free BceAB-S complex. Although the two states could be readily classified in 3D classification, the overall structures are highly similar (RMSD ~0.6Å across TM region).

**Title**: Movie S2

**Description**: Movies showing the motion observed in the nucleotide-free BceAB-S complex as revealed by 3DVA in CryoSPARC. The first three principal components of motion are shown, demonstrating a high degree of conformational flexibility in the BceS soluble domains relative to the rest of the complex.

**Title**: Movie S3

**Description**: Morph between nucleotide-free and ATP𝛾S bound BceAB-S structures. The interpolation between structures demonstrates that movement of BceA and BceS in response to ATP𝛾S binding appears to be coordinated. As the BceA subunits capture ATP𝛾S and move toward a more symmetrical configuration the soluble regions of BceS move in concert with BceA.
